# The Distribution of Fitness Effects of Beneficial Mutations in *Pseudomonas aeruginosa*

**DOI:** 10.1371/journal.pgen.1000406

**Published:** 2009-03-06

**Authors:** R. Craig MacLean, Angus Buckling

**Affiliations:** Department of Zoology, University of Oxford, Oxford, United Kingdom; Université Paris Descartes, INSERM U571, France

## Abstract

Understanding how beneficial mutations affect fitness is crucial to our understanding of adaptation by natural selection. Here, using adaptation to the antibiotic rifampicin in the opportunistic pathogen *Pseudomonas aeruginosa* as a model system, we investigate the underlying distribution of fitness effects of beneficial mutations on which natural selection acts. Consistent with theory, the effects of beneficial mutations are exponentially distributed where the fitness of the wild type is moderate to high. However, when the fitness of the wild type is low, the data no longer follow an exponential distribution, because many beneficial mutations have large effects on fitness. There is no existing population genetic theory to explain this bias towards mutations of large effects, but it can be readily explained by the underlying biochemistry of rifampicin–RNA polymerase interactions. These results demonstrate the limitations of current population genetic theory for predicting adaptation to severe sources of stress, such as antibiotics, and they highlight the utility of integrating statistical and biophysical approaches to adaptation.

## Author Summary

Adaptation by natural selection depends on the spread of novel beneficial mutations, and one of the most important challenges in our understanding of adaptation is to be able to predict how beneficial mutations impact fitness. Here, we investigate the underlying distribution of fitness effects of beneficial mutations that natural selection acts on during the evolution of antibiotic resistance in the opportunistic human pathogen *P. aeruginosa*. When the fitness of the wild type is high, most beneficial mutations have small effects. This finding is consistent with existing population genetic models of adaptation based on statistical theory. When the fitness of the wild type is low, most beneficial mutations have large effects. This distribution cannot be explained by population genetic theory, but it can be readily understood by considering the biochemical basis of resistance. This study confirms an important prediction of population genetic theory, and it highlights the need to integrate statistical and biochemical approaches to adaptation in order to understand evolution in stressful environments, such as those provided by antibiotics.

## Introduction

**Figure 1:**
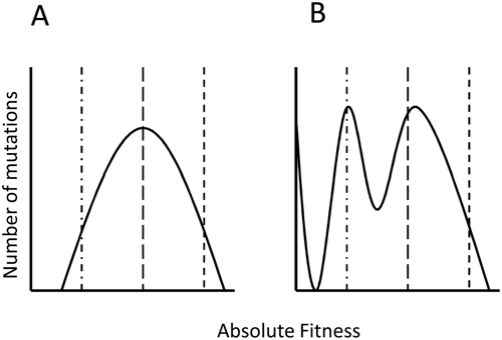
A schematic of the distribution of fitness effects of beneficial mutations. This figure is a schematic showing how the distribution of fitness effects of beneficial mutations can change depending on the absolute fitness of the wild type (shown with vertical lines) in two different arbitrary distributions of mutational effects on fitness. Note that the distribution of fitness effects of beneficial mutations is very similar in both of these distributions when the fitness of the wild-type is high (short dashed line).

Adaptation by natural selection ultimately depends on the spread of novel beneficial mutations that increase fitness. Can we predict the fitness effects of beneficial mutations? Gillespie[1],[2] argued that extreme value theory (EVT) provides a simple answer to this question: the tails of all-Gumbel type distributions (a very flexible type of distribution that includes many familiar distributions, including the normal) are exponential. As such, the fitness effects of beneficial mutations will be exponentially distributed provided that the fitness of the wild-type is high enough so that beneficial mutations are drawn from the extreme tail of the distribution of fitness effects of mutations. It is however unclear how robust this theory is with respect to the fitness of the population prior to selection. As the absolute fitness of the wild-type decreases (for example, because of environmental change), a larger proportion of single mutations will increase fitness. Beneficial mutations will therefore no longer be drawn exclusively from the tail of the distribution, hence the exponential distribution may no longer apply (Figure 1).

In this paper, we investigate how the distribution of fitness effects of beneficial mutations changes with the fitness of the wildtype population using the evolution of antibiotic resistance in the opportunistic pathogen *Pseudomonas aeruginosa* as a model system. Experimental studies of the underlying distribution of fitness effects of beneficial mutations[3],[4],[5],[6] have lagged behind the theory, both because beneficial mutations are exceedingly rare and because beneficial mutations of small effect are less likely to reach appreciable frequencies in populations because of the combined effects of drift[7] and competition between independent mutations[8]. To overcome these limitations, we used a fluctuation test to isolate clones of the bacterium *Pseudomonas aeruginosa* with mutations in the β-subunit of RNA polymerase (*rpoB*) that are beneficial in the presence of the drug rifampicin [9],[10],[11]. Our experimental design ensured that we obtained an unbiased sample of all beneficial mutations. First, we isolated mutants from populations that were propagated in culture media lacking rifampicin, implying that we isolated beneficial mutations prior to any selection for rifampicin resistance. Second, we experimentally prevented competition (ie clonal interference[8]) among independently derived beneficial mutations by randomly choosing independent mutants. Third, we ensured all mutations included in the analyses were unique by sequencing *rpoB* of the mutants. To test the hypothesis that the fitness effects of beneficial mutations are exponentially distributed, we used log-likelihood tests that have been specifically developed to test this hypothesis using this experimental design[12].

Using this approach, we show that the distribution of fitness effects of beneficial mutations is variable: under conditions where the fitness of the wild-type is high, the fitness effects of beneficial mutations are exponentially distributed, as predicted by theory. However, when the fitness of the wildtype is low, the data may no longer fit an exponential distribution because many beneficial mutations have large effects on fitness. We show that this non-exponential distribution of fitness effects emerges as a direct consequence of the molecular interactions that are under selection in this system and we argue that existing theory on the fitness effects of beneficial mutations cannot be applied to understand adaptation to novel stressful environments, such as those provided by antibiotics.

## Results/Discussion

**Figure 2:**
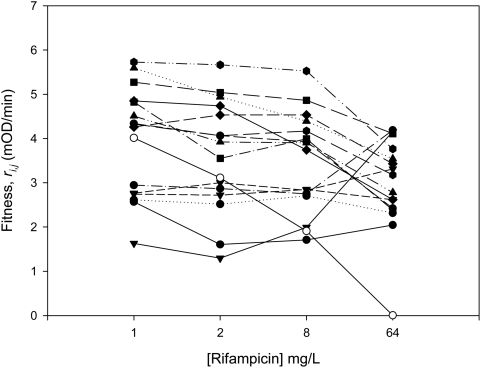
Fitness effects of beneficial mutations. Plotted points show the fitness, *r*, of the wild-type (open symbol) and beneficial mutations (filled symbols) at different concentrations of rifampicin. Fitness was estimated as the mean growth rate of 12 to 18 cultures of each strain at each concentration of rifampicin (standard errors not shown).

**Figure 3:**
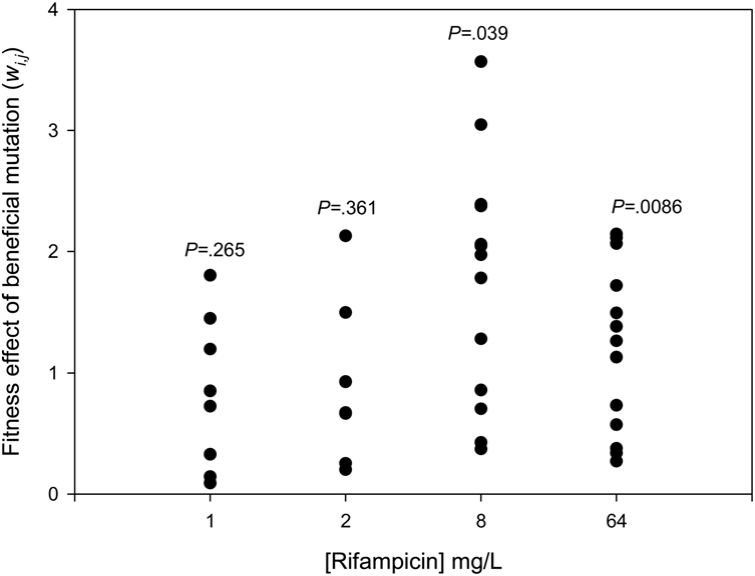
Distribution of fitness effects of beneficial mutations. Plotted points show *w_i,j_*, the difference in fitness between beneficial mutations (i.e. those mutations that give higher fitness than the ancestral clone) and the least-fit beneficial mutation at each concentration of rifampicin. *P* values show the probability that the observed distribution of fitness effects of beneficial mutations is exponential (see table 2 for further statistical details).

To investigate how the distribution of fitness effects of beneficial mutations changes with the fitness of the wild-type, we measured the fitness of beneficial mutations isolated at a high concentration of rifampicin (Table 1) and the wild-type across a gradient of rifampicin concentrations (Figure 2). At low concentrations of rifampicin (1–2 ug/mL), the fitness of the wildtype is high and we cannot reject the null hypothesis that the fitness effects of beneficial mutations are exponentially distributed, as determined by a likelihood-ratio test (Figure 3, Table 2). However, at high concentrations of rifampicin (>2 ug/mL), the fitness of the wild-type is low and the fitness effects of beneficial mutations are not exponentially distributed (Figure 3, Table 2).

**Table 1:** Beneficial mutations.

Mutation in *rpoB*	Amino acid change
1552CGC1553	+P518 (insertion between 517 and 518)
A1553G	Q518R
A1553T	Q518L
A1562G	D521G
A1562T	D521V
A1592G	H531R
A1592T	H531L
A455G	Q152R
A455T	Q152L
C1550T	S517L
C1552A	Q518K
C1563G	D521E
C1591T	H531Y
C1607T	S536F
C1706T	P569L

This table shows the beneficial mutations in *rpoB* that we identified in this study. We used a fluctuation test to isolate a total of 80 independent clones carrying beneficial mutations and sequencing revealed that each one of the clones carried one of 15 non-synonymous mutations in *rpoB*. We used one representative clone carrying each of these beneficial mutations in our experiments.

**Table 2:** Testing the exponential distribution.

[Rifampicin] mg/L	Relative growth rate of wild type (*r_PAO1,j_*)	−2logΛ	*n*	*P*
1	.79	3.47	9	.265
2	.70	2.4	9	.361
8	.38	7.62	14	.039
64	.001	10.71	15	.0086

We used a likelihood ratio test to test the null hypothesis that the fitness effects of beneficial mutations are exponentially distributed at different concentrations of rifampicin (see methods for details). *n* gives the number of mutations that increase fitness at each dose of antibiotic. The growth rate of the wild-type strain was standardized relative to the growth rate of this clone in antibiotic-free medium.

One limitation of this study is that our power to test the null hypothesis is weakest under conditions where the fitness of the wild-type is high, because only half of the mutants that we isolated increase fitness at low concentrations of rifampicin. Given that we had to sample 80 mutants in order to identify 15 beneficial mutations (this saturation effect is in part attributable to a strong mutational bias towards two mutations), it unlikely that increasing the sample size of our study would have substantially increased the power of our analysis. It is also important to note that this limitation is not unique to this study: beneficial mutations are rare events, and all other comparable experimental evolution studies are based on a similar sample size of beneficial mutations[4],[13].

**Figure 4:**
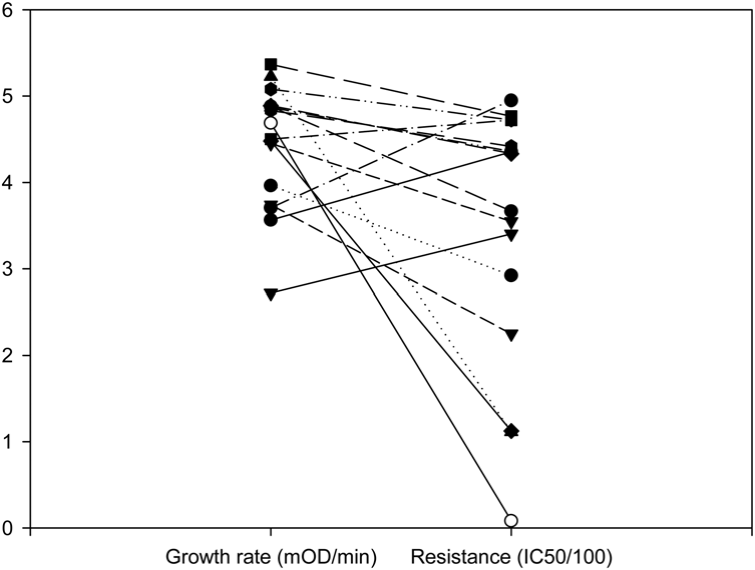
Phenotypic traits under selection. Plotted points show the rifampicin resistance and growth rate in media lacking rifampicin of beneficial mutation (filled symbols) and the wildtype (open symbols).

To gain insight into the mechanistic basis of fitness, we measured both the growth rate in the absence of antibiotics and the degree of rifampicin resistance for each beneficial mutation (Figure 4). At low concentrations of rifampicin (ie 1–2 ug/mL), selection for high levels of resistance is weak, and fitness is highly correlated with growth rate in the absence of antibiotics (*r* = .86–.9, *P*<.0001). The genetic variation in growth rate in the absence of antibiotics generated by spontaneous mutation is normally distributed (Figure 3; *W* = .92, *P* = .21), hence the fitness effects of beneficial mutations are exponentially distributed because only mutations in the right tail of the distribution (ie those mutations that are associated with a low cost of resistance[14]) are beneficial at low concentrations of rifampicin. At high concentrations of rifampicin, selection for resistance is strong and the fitness effects of beneficial mutations no longer fit an exponential distribution because most beneficial mutations had large effects on resistance and, therefore, fitness at high concentrations of rifampicin (Figure 4).

**Figure 5:**
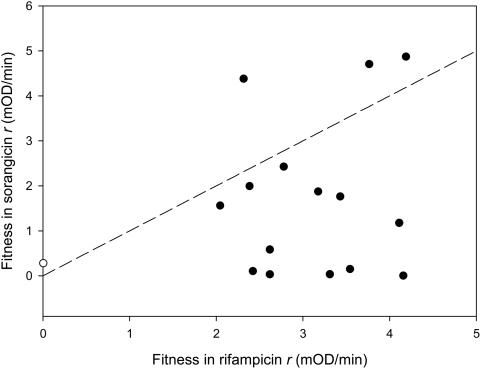
Fitness in the presence of sorangicin. This figure shows the fitness of beneficial mutations (filled symbols) and the ancestral clone (open symbol) at 64 mg/L rifampicin (n = 18 replicates/genotype) and 20 mg/L sorangicin (n = 6 replicates/genotype). The dashed line is a plot of y = x.

The large effect of beneficial mutations on resistance is consistent with the molecular interactions that occur between rifampicin and RNA polymerase. Structural studies have shown that rifampicin binds to a small, highly conserved pocket of the β-subunit of RNAP and only 12 amino acid residues are involved in direct interactions with rifampicin[9],[11]. Mutations at these residues cause a large increase in resistance (mean IC_50_ = 423 ug/mL, s.e = 25 ug/mL, n = 10). Residues that surround the binding pocket interact only indirectly with rifampicin, and it has been argued that resistance arises at these residues due to amino acid changes that alter the folding of the protein in the binding pocket. We identified only a small number of beneficial mutations (n = 4) in residues that are involved in indirect Rif-RNAP interactions and mutations at these residues give rise to intermediate levels of rifampicin resistance (mean IC_50_ = 197 ug/mL, s.e = 40 ug/mL, n = 4). This biophysical approach to understanding the effects of beneficial mutations suggests that the data may no longer fit an exponential distribution because of the high specificity of interactions between rifampicin and RNA polymerase: changes to the majority of amino acids that are involved in rifampicin-RNAP interactions results in large increases in resistance and, therefore, large increases in fitness at high concentrations of rifampicin. To test this hypothesis further, we assayed fitness in the presence of sorangicin[15], an antibiotic that has been shown to bind to the same domain of RNAP as rifampicin and share the same mode of action, inhibition of transcription initiation[16]. The biochemical difference between these antibiotics comes from the fact that sorangicin has a much higher conformational flexibility than rifampicin [16]. The fitness consequence of this difference is that many mutations that give a large increase in fitness under high concentrations of rifampicin give only a small increase in fitness in the presence of an equivalent dose of sorangicin (Figure 4), and the observed distribution of fitness effects of beneficial mutations does not differ significantly from the exponential (Figure 5, −2logΛ = 5.72, n = 9, *P* = .09).

It is important to note that these results do not necessarily imply that the distribution of fitness effects of mutations that are beneficial in the presence of sorangicin is universally exponential. Most mutations that increase sorangicin resistance do so by altering membrane permeability, instead of altering the structure of RNAP[16], but unfortunately the mutations that are responsible for this decrease in permeability to sorangicin are not known and we are therefore unable to measure the underlying distribution of fitness effects of mutations that are beneficial in the presence of sorangicin. Instead, our interpretation of this result is that it provides a clear demonstration that the high-affinity interactions that occur between rifampicin and RNAP are ultimately responsible for the non-exponential distribution of fitness effects of beneficial mutations at high concentrations of rifampicin.

### Conclusion

The variability in the distribution of fitness effects of beneficial mutations in this study is consistent with population genetic theory. When the fitness of the wild-type is high, beneficial mutations can be viewed from a statistical perspective as representing draws from the extreme tail of the distribution of fitness effects of mutations, hence the fitness effects of beneficial mutations will be exponentially distributed. However, EVT does not specify how high fitness has to be in order for this theory to apply. In our experimental system, this distribution held over a wide range of parameter space: we failed to detect significant deviations from the exponential distribution when the fitness of the wild-type was reduced by 20–30%. When the fitness of the wild-type is low, statistical theory does not make any predictions regarding the form of the distribution of fitness effects of beneficial mutations and hence there is no reason to expect an exponential distribution. Comparable experimental studies in viral systems are in agreement with this idea: Sanjuan and colleagues [4] found that the fitness effects of beneficial mutations are exponentially distributed in VSV under conditions where the fitness of the wild-type is high, while Rokyta *et al.* [3] found that the fitness effects of beneficial mutations that allow phage to attack novel hosts (ie hosts that are inaccessible to a WT virus) are not exponentially distributed. Note that in these and our experiments, the data may stop fitting the exponential distribution under conditions of low wildtype fitness not only because EVT, by definition, no longer applies, but also because the underlying mutational distributions may vary with environmental conditions.

Despite our lack of certainty of the statistical explanation for the observed distribution when the fitness of the wild-type is low, we have a good molecular mechanistic explanation (in retrospect we may have been able to predict this distribution *a priori*). Specifically, the distribution is biased towards mutations of large effect as a result of the high specificity of interactions between rifampicin and RNA polymerase that arises from the low conformational flexibility of rifampicin. Antibacterial and antiviral drugs are usually involved in highly specific interactions with their target proteins [17], suggesting that a bias towards mutations of large effect may be a general feature of adaptation to antibiotics [17],[18] and other situations when high-specificity protein-ligand interactions are under strong selection, for example during host-parasite interactions[19] or when enzymes are selected to recognize novel substrates[20],[21].

Recently, there has been considerable interest among population geneticists in developing general models of adaptation based on the statistical properties of extreme events. Our work highlights both the strengths and limitations of this approach and we suggest that the development of a complete theory of adaptation will require integrating molecular biology, in order to be able to predict the impact of mutations on fitness, and statistical approaches to adaptation, to be able to understand how natural selection samples the distribution of fitness effects of beneficial mutations during adaptive walks.

## Materials and Methods

### Isolation of Beneficial Mutations

A single clone of *Pseudomonas aeruginosa* PAO1 was inoculated into 5 ml of M9KB medium that was incubated overnight at 37 C with constant shaking (150 rpm). This overnight culture was diluted down 10^−6^ into fresh M9KB and 120 uL aliquots of this diluted culture were used to setup 480 cultures on 5 96 well microplates. These cultures were incubated overnight at 37 C without shaking. To isolate beneficial mutations, 5 uL of each of the 480 overnight cultures was plated out on M9KB supplemented with 62.11 ug/mL of rifampicin, the minimal concentration required for complete inhibition of growth of the wildtype strain. To isolate beneficial mutations, we isolated a single colony from each of the first 80 cultures that gave samples containing exactly 1 colony on agar plate containing rifampicin.

### Sequencing

To determine the mutations underlying adaptation, we sequenced the *rpoB* gene in each of the 80 colonies that we isolated in our fluctuation test. Genomic DNA was isolated from each colony using a Wizard Genomic DNA extraction kit (Promega, UK) as per the manufacturer's instructions. Our sequencing strategy was to first sequence a highly-conserved domain of *rpoB* that is known to be important for rifampicin resistance in all 80 clones. This region was amplified with primers rpoB_fwd (5′-GTTCTTCAGCGCCGAGCG-3′) and rpoB_rev (5′-GCGATGACGTGGTCGGC-3′) that amplify the region of the *rpoB* gene between nucleotides 1178 and 1864. Reaction mixtures consisted of BIOTAQ polymerase (Bioline, UK), 1 mM dNTPs, 16 nM (NH_4_)_2_SO_4_, 62.5 mM Tris-HCL (pH 8.8), .01% Tween 20, 2 mM MgCl_2_, and each primer at a concentration of .2pM. Amplification reactions were carried out as follows: 94 C for 5 minutes, followed by 35 cycles of 94 C for 30 seconds, 60 C for 30 seconds, and 72 C for 1 minute, followed by a final incubation at 72 C for 10 minutes. PCR products were purified using a MultiScreen PCR_96_ filter plate (Millipore, UK) as per the manufacturer's instructions. Purified PCR products were sequenced with both forward and reverse primers using BigDye 3.1 sequencing (Applied Biosystems International) followed by ethanol/EDTA precipitation of sequencing products. In all cases, this strategy identified either a single mutation in this region of *rpoB* or no mutations.

Clones that lacked a mutation in the highly conserved domain of *rpoB* were subsequently sequenced for a second region that has previously been implicated in rifampicin resistance spanning nucleotides 1 to 1012 of the *rpoB* gene. This region was amplified and sequenced with primers rpoB_up (5′–ATGGCTTACTCATACACTGAG-3′) and rpo_B1 (5′-CTCGATGCG CACGACCTG-3′). The protocol was the same as described above, except that the annealing temperature used in the PCR reactions was 54 C instead of 60 C. We idenfied a single mutation in this region in all of the clones that did not contain a mutation in the highly conserved domain of *rpoB*. As a further control, we sequenced the entire *rpoB* gene in six randomly chosen clones. We failed to detect any second site mutations in *rpoB* using this approach.

### Fitness Assay

To assay fitness, we estimated the growth rate, *r*, of each beneficial mutation and wildtype PAO1 at 4 different concentrations of rifampicin (0,1,2,8, and 64 mg/L). Pre-assay overnight cultures of each mutant were prepared by growth in M9KB. These cultures were then diluted 100 fold into fresh culture medium and we measured the growth rate of each culture using an automated microplate reader by taking hourly measurements of optical density at 600 nm (OD_600_) over a period of approximately 12 hours. All incubations were carried out at 37 C. Assays at low concentrations of rifampicin (0,1,2,8 mg/L) were carried out with 12 fold replication and assays at high concentrations of rifampicin (64 mg/L) were carried out with 18 fold replication. A further assay was carried out using the same method to measure fitness in the presence of sorangicin (20 ug/mL). OD_600_ is proportional to the log of cell density, and the slope of OD_600_ against time (mOD/min) in exponential growth phase therefore provides an estimate of *r*, the growth rate of the bacterial clone, such that *r_i,j_* is the growth rate to the *i* ^th^ genotype at the *j* ^th^ concentration of rifampicin.

### Statistical Analysis

To test the hypothesis that the fitness effects of beneficial mutations are exponentially distributed, we used a likelihood ratio test developed by Beisel and colleagues [12]. According to EVT, there are three limiting tail distributions, the Fréchet (which has a heavier-than-exponential tail), the Gumbel (which has an exponential tail), and the Weibull (right-truncated). The tails of all three of EVT domains can be described by the generalized Pareto distribution (GPD), which has a cumulative distribution function given by:
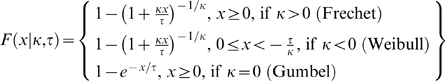
with shape parameter κ and scale parameter τ. One very interesting property of the GPD is that the shape parameter is threshold-independent. This property of the GPD is critical, because it implies that it is possible to account for any potential bias against detecting mutations of small beneficial effect by simply re-scaling fitness data so that the fitness of beneficial mutations is expressed relative to the least-fit beneficial mutation instead of the wild-type. To take advantage of this property, we estimated the fitness of each beneficial mutation as as *w_i,j_* = *r_i,j_*−*r_1,j_* where *w_i,j_* is the fitness of the *i* ^th^ beneficial mutation in the *j* ^th^ environment, *r_i,j_* is the growth rate of the *i* ^th^ beneficial mutation in the *j* ^th^ environment, and *r_1,j_* is the growth rate of the least fit beneficial mutation in the the *j* ^th^ environment (ie the beneficial mutation that has the smallest increase in growth rate relative to the rifampicin sensitive clone in the *j* ^th^ environment).

The likelihood ratio test developed by Beisel and colleagues calculates −2logΛ, negative twice the difference in log-likelihood between two statistical models, one model in which the shape parameter of the GPD is set to 0 and the other in which the scale parameter of the GPD is free to vary (ie H_0_:κ = 0, H_A_: κ≠0). *P* values were calculated by performing 10000 parametric bootstrap replicates using a software package (EVDA) developed for R (software available at http://www.webpages.uidaho.edu/˜joyce/Lab20Page/Computer-Programs.html). This test is potentially sensitive to measurement error, but the accuracy of our fitness measurements was high enough (Average CV = 19.8%) that measurement error should not inflate the probability of making a type I error [12].

### Inhibition Assays of *rpoB* Mutants

To measure the resistance conferred by *rpoB* mutations, we assayed growth in media containing rifampicin at different concentrations. Pre-assay cultures of *rpoB* mutants and PAO1 were prepared by overnight growth of freezer cultures in M9KB at 37 C. These cultures were then diluted 2.5×10^−5^ into M9KB, or M9KB supplemented with rifampicin at the following concentrations (all in ug/mL): 0, 3.9, 7.8, 15.6, 31.3, 62.5, 125, 250, 500, and 1000. Assay cultures were incubated at 37 C and we measured the optical density of cultures after exactly 24 hours of incubation (+/−10 minutes) at 600 nM using an automated microplate reader. We assayed the resistance of 12 cultures of each of the *rpoB* mutants that we identified and 12 replicates of PAO1. Resistance was calculated as IC_50_, the concentration of rifampicin necessary to cause a 50% reduction in optical density, using the following regression model: 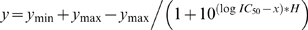, where *y* is optical density, measured in absorbance units, *x* is the concentration of rifampicin, measured in ug/mL, and *H* is parameter that estimates the rate of decay in optical density with increasing rifampicin concentration.
